# Electromagnet Weight Reduction in a Magnetic Levitation System for Contactless Delivery Applications

**DOI:** 10.3390/s100706718

**Published:** 2010-07-09

**Authors:** Do-Kwan Hong, Byung-Chul Woo, Dae-Hyun Koo, Ki-Chang Lee

**Affiliations:** 1 Electric Motor Research Center, Korea Electrotechnology Research Institute, Boolmosangil 70, Changwon, 641-120, Korea; E-Mails: bcwoo@keri.re.kr (B.-C.W.); dhk371@keri.re.kr (D.-H.K.); 2 School of Mechanical Engineering, Pusan National University, Geumjeong-gu, Busan, 609-735, Korea; E-Mail: leekc@pusan.ac.kr (K.-C.L.)

**Keywords:** optimum design, response surface methodology (RSM), levitation control, kriging interpolation method, design of experiments (DOE), electromagnet

## Abstract

This paper presents an optimum design of a lightweight vehicle levitation electromagnet, which also provides a passive guide force in a magnetic levitation system for contactless delivery applications. The split alignment of C-shaped electromagnets about C-shaped rails has a bad effect on the lateral deviation force, therefore, no-split positioning of electromagnets is better for lateral performance. This is verified by simulations and experiments. This paper presents a statistically optimized design with a high number of the design variables to reduce the weight of the electromagnet under the constraint of normal force using response surface methodology (RSM) and the kriging interpolation method. 2D and 3D magnetostatic analysis of the electromagnet are performed using ANSYS. The most effective design variables are extracted by a Pareto chart. The most desirable set is determined and the influence of each design variable on the objective function can be obtained. The generalized reduced gradient (GRG) algorithm is adopted in the kriging model. This paper’s procedure is validated by a comparison between experimental and calculation results, which shows that the predicted performance of the electromagnet designed by RSM is in good agreement with the simulation results.

## 1. Introduction

**Figure 1.**
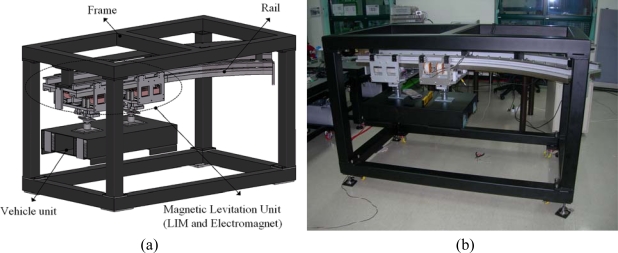
Magnetic levitation system prototype for contactless delivery application: (a) 3D modeling (b) prototype.

Electromagnetically levitated and guided systems with linear motor propulsion are commonly used in the field of people transport vehicles, tool machines and conveyor systems because of their silent and non-contact motion [1–3]. Passive guidance controls are normally used in real implementations of Maglev systems with linear induction motor propulsion to reduce construction costs and complexity. The Japanese HSST and the English BAMS (Birmingham Airport Maglev System) are well-known cost-effective transport systems based on linear induction motor propulsion and magnetic levitation. In both systems, the guidance force needed to keep the vehicles on the track is obtained by the levitation electromagnets with the help of particular shapes of the rail and clever placement of the electromagnets about it. In Maglev transport systems where levitation magnets are on moving parts, the design of levitation electromagnet is the most important factor. It can account for a considerable part (10%) of the whole vehicle weight, which can largely affect weight and stability of the magnetic levitation system. Optimum plans for the weight reduction of the levitation electromagnet are consequently desirable [4–7]. In this paper, an optimal design procedure was required in order to develop a magnetic levitation system for a contactless delivery application shown in Figure 1. As a first step, the design goal was to reduce the weight of the electromagnet of the magnetic levitation system with the constraint of normal force, considering the initial model. At a second step, the most effective design variables and their levels should be determined and be arranged in an orthogonal array table.

**Figure 2.**
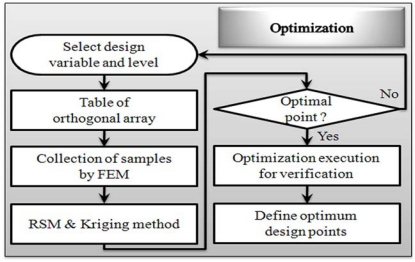
Optimization flow chart.

**Figure 3.**
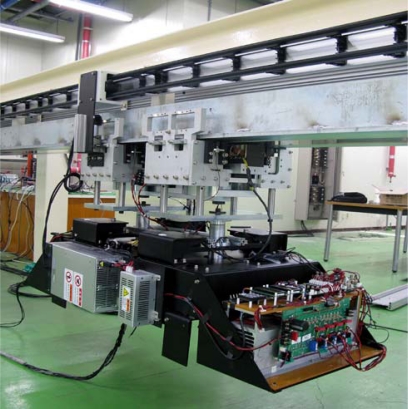
Application prototype.

Response Surface Methodology (RSM) is generally used with two or three design variables, however we have seven design variables, so a mixed orthogonal array table was utilized. The reason for this is that a mixed orthogonal array table is an efficient way to study the effect of several design variables simultaneously with a number small of experiments and to plan matrix simulation trials. For each design variable combination the response value is determined by the 2D and 3D Finite Element Method (FEM). In this paper we use the reduced gradient algorithm, which can lead to the selection of the most desired set of variables. Figure 2 shows optimization procedure flow chart of RSM and kriging interpolation method. Figure 3 shows the application prototype model for applying the contactless delivery application. The response is determined and we evaluate the influence of each design variable on the objective function. Based on this method the weight of the optimized electromagnet could be reduced by 11.412% and normal force improved by 7.754%, compared to the initially designed electromagnet.

## 2. Passive Guidance Control and Optimization of Electromagnet

**Figure 4.**
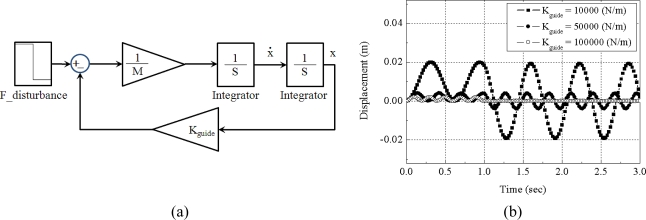
Lateral position model for electromagnets under constant levitation control: (a) passive guidance model; (b) simulation result for a 100 N disturbance.

Let’s assume that the guidance forces are generated by closed-loop control of levitation electromagnets. The lateral response of the electromagnet due to the airgap control is a force increasing with the lateral offset X. This force is almost a linear function of the lateral offset of the electromagnets. Then we can imagine that the electromagnet will move laterally as a mass bound by a spring. As we know, a spring working on a mass is a mechanical resonant system, whose resonance frequency is given by:

(1) $$f_{\mathrm{resonance}}=\frac{1}{2\pi }\sqrt{\frac{K_{\text{guide}}}{M}}$$

where the M is the sum of the masses of the electromagnet and of part of the carried vehicle. We can easily imagine that any external action will cause a non-damped oscillating response of the lateral position. The value of k_guide_ can be designed to keep the disturbance from causing an excessive displacement. An example of the design problem is shown in Figure 4.

A total mass of 100 kg is assumed to be levitated with a constant gap. The sum of lateral position stiffness of the levitation magnets is set at 10,000 N/m, 50,000 N/m and 100,000 N/m, respectively. The lateral disturbance forces are assumed to be a constant value of 100 N for about 1 second. The simulation result shows that if the lateral position stiffness remains higher than 50,000 N/m, the lateral position deviation is smaller than 5 mm under constant 100 N disturbance.

**Figure 5.**
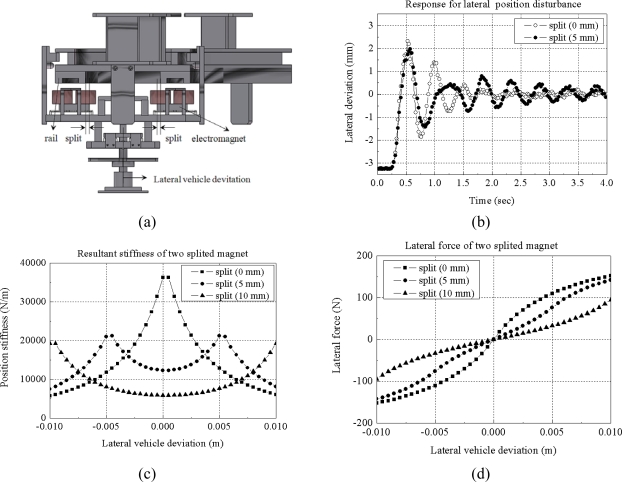
Lateral position response of magnetically sprung mass under position disturbance: (a) experimental model; (b) lateral position response (experiment); (c) lateral position stiffness response (simulation); (d) lateral force response (simulation).

In the experiment, a total mass of 200 kg including four levitation electromagnets is levitated under small deviation of electromagnet placement conditions, as shown in Figure 5. That is, a mass of 50 kg is levitated by one electromagnet. In the experiment, levitation magnets are controlled to maintain a constant gap length of 5 mm. A position disturbance of 3.2 mm is applied at first and then removed. The lateral response of the experiment is shown in Figure 5(b). We can infer that if two electromagnets are positioned with small deviation from the original rail position, the guidance forces generated each electromagnet can be larger, but the resultant force becomes smaller because of the differential actuation scheme, so the position stiffness become smaller, the natural frequency becomes smaller and the lateral deviation becomes larger as the deviation of magnets becomes larger. Therefore, no-split positioning of electromagnets is better for lateral performance.

## 3. Optimum Theory

### 3.1. Response Surface Methodology

The RSM method can be readily adapted to develop an analytical model for a complex problem. With this analytical model, an objective function with constraints can be easily created and evaluated, and computation time can be saved. A polynomial approximation model is commonly used for a second-order fitted response (*u*) and can be written as follows:

(2) $$\begin{array}{l} u=\beta_{0}+\sum_{j=l}^{k}\beta_{j}x_{jj}+\sum_{j=1}^{k}\beta_{jj}x_{j}^{2}+\sum_{j=l}^{k}\beta_{ij}x_{i}x_{j}+\varepsilon \\ \beta :\text{regression coefficients},\;\;\;x:\text{design variables}\\ \varepsilon :\text{random error},\;\;k:\text{number of design variables} \end{array}$$

The least squares method is used to estimate unknown coefficients. Matrix notations of the fitted coefficients and the fitted response model should be as shown in equation (3):

(3) $$\hat{\beta }={\left(X^{\prime }X\right)}^{-1}X^{\prime }u\;,\;\hat{u}=X\hat{\beta }$$

where β̂ is a vector of the unknown coefficients which are usually estimated to minimize the sum of the squares of the error term. It should be evaluated at the data points. RSM can be applied in connection with FEM and the response actually represents FEM result.

### 3.2. Kriging Interpolation Method

Kriging is a method of interpolation named after a South African mining engineer D. G. Krige, who developed the technique while trying to increase accuracy in predicting ore reserves. In the kriging model, the global approximation model for a response y(**x**) is represented as:

(4) y(x)=β+ν(x)

where **x** is the design variable vector, *β* is a constant, and ν*(***x***)* is the realization of a stochastic process. In Equation (4), ν*(***x***)* has the mean zero, variance σ*^2^*, and non-zero covariance. The weight of electromagnet *Weight_total_* is replaced by *y(***x***)* to make a surrogate approximation model. Let $\hat{y(x)}$ be an approximation model. Hereafter, ^ means the estimator. When the mean squared error between *y(***x***)* and $\hat{y(x)}$ is minimized, $\hat{y(x)}$ becomes:

(5) $$\hat{y(x)}=\hat{\beta }+r^{T}(x)R^{-1}\left(y-\hat{\beta }q\right)$$

where **r** is the correlation vector, **R** is the correlation matrix, **y** is the observed data and **q** is the unit vector. The definitions of **R** and **r** are well explained in Refs [8,9].

The unknown correlation parameters of *θ_1_*, *θ_2_*,…,*θ_n_* defined in **R** are calculated from the formulation according to:

(6) $$\mathrm{maximize}-\frac{\left[n_{s}\ln\left(\hat{\sigma^{2}}\right)+\ln|R|\right]}{2}$$

where *θ_i_* (i = 1,2,…,n) > 0. In this study, a GRG (generalized reduced gradient) algorithm built in the Excel program was utilized to determine the optimum parameters.

## 4. Optimum Design

### 4.1. Metamodel and Design Variable

Optimization formulation:

(7) $$\text{Minimize}:{\mathrm{Weight}}_{\mathrm{total}}\left({\mathrm{dv}}_{1},\ldots ,{\mathrm{dv}}_{7}\right)$$

(8) $$\text{Subject to}\;\;\;F_{\mathrm{normal}}\left({\mathrm{dv}}_{1},\;\ldots ,\;{\mathrm{dv}}_{7}\right)\geq 611.689$$

**Figure 6.**
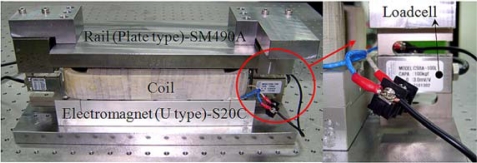
Prototype of the electromagnet.

**Figure 7.**
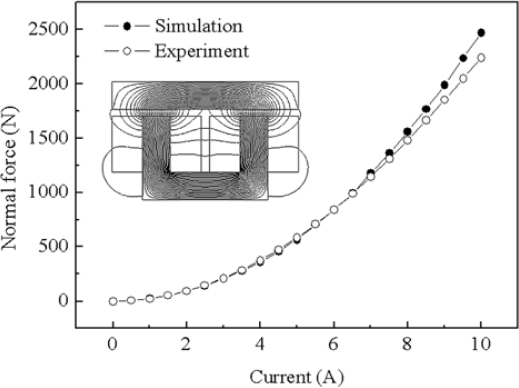
Comparison of FEM and experiment (reference model).

Figure 6 shows the prototype electromagnet of the reference model which is analyzed. The comparison of the static force obtained from FEM simulation and experimental test is shown in Figure 7. From the results, the use of FEM is validated, as it can be observed, since the comparison of normal force by simulation and experiment test shown in Figure 7 displays good agreement. Selection of the design variables is very important setup in optimization procedure.

**Figure 8.**
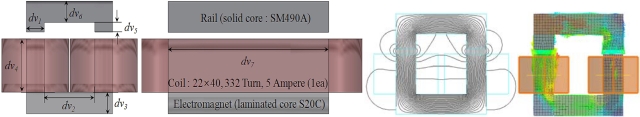
Design variables and flux pattern (2D and 3D FEM).

**Figure 9.**
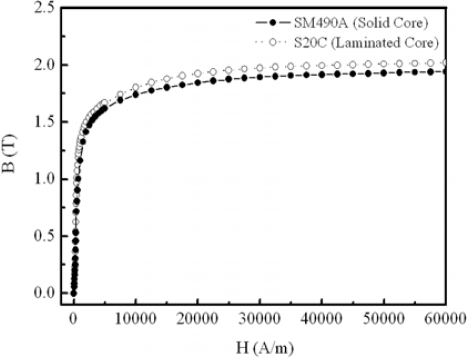
Magnetization curve of the core material used.

Seven dimensions are selected as the design variables as shown in Figure 8, which shows the flux density vector in electromagnet of 2D and 3D FEM. The magnetization characteristics of the S20C, SM490A material is as shown in Figure 9. Table 1 shows the design variables and levels.

### 4.2. Response Surface Methodology

Table 2 shows the table of mixed orthogonal array and simulation results by 2D FEM. The mixed orthogonal array table is determined by considering the number of the design variables and each level of them. After getting experimental data by FEM, the function to draw a response surface is extracted. In order to determine equations of the response surface for each response value (weight, normal force), several experimental designs have been developed to establish the approximate equation using the smallest number of experiments. The purpose of this paper is to minimize the objective function (*Weight_total_*) with constraints of normal force (*F_normal_*). The two fitted second order polynomial of objective functions for the seven design variables are as follows:

(9) $$\begin{array}{l} \begin{array}{lll} F_{\mathrm{normal}} & = & 148.75+26.1{\mathrm{dv}}_{1}-2.41{\mathrm{dv}}_{2}-10.02{\mathrm{dv}}_{3}-32.18{\mathrm{dv}}_{4}-1.63{\mathrm{dv}}_{5}+35.16{\mathrm{dv}}_{6}\\ & & +3.01{\mathrm{dv}}_{7}-7.14E-2{\mathrm{dv}}_{1}^{2}+1.3E-3{\mathrm{dv}}_{2}^{2}+0.23{\mathrm{dv}}_{3}^{2}+0.35{\mathrm{dv}}_{4}^{2}+5.56E-2{\mathrm{dv}}_{5}^{2}\\ & & -0.9{\mathrm{dv}}_{6}^{2}-3.72E-4{\mathrm{dv}}_{7}^{2} \end{array}\\ \begin{array}{lll} {\mathrm{Weight}}_{\mathrm{total}} & = & 8.765+0.212{\mathrm{dv}}_{1}-0.248{\mathrm{dv}}_{2}-0.154{\mathrm{dv}}_{4}-0.488{\mathrm{dv}}_{4}-0.113{\mathrm{dv}}_{5}+0.264{\mathrm{dv}}_{6}\\ & & +0.065{\mathrm{dv}}_{7}+1.84E-3{\mathrm{dv}}_{1}^{2}+2.8E-3{\mathrm{dv}}_{2}^{2}+6.54E-3{\mathrm{dv}}_{3}^{2}+5.95E-3{\mathrm{dv}}_{4}^{2}\\ & & +7.58E-3{\mathrm{dv}}_{5}^{2}-4.05E-3{\mathrm{dv}}_{6}^{2}+2.64E-6{\mathrm{dv}}_{7}^{2} \end{array} \end{array}$$

**Figure 10.**
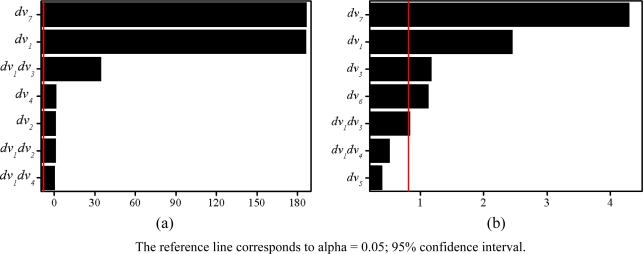
Pareto chart of the standardized effects (alpha=0.05) (a) Normal force response (b) Total weight response.

The adjusted coefficients of the multiple determination R^2^_adj_ for normal force and weight are *weight_total_* (99%) and *F_normal_* (100%). Normal force and weight of experiment result according to change of the design variables are shown in Table 2. As many parameters are defined as design variables, a large simulation time is required due to the large number of required experiments. Therefore, it is necessary to establish the significant parameters to investigate the influence on the design result. The Pareto chart of normal force and weight shows the magnitude and importance of an effect. This chart displays the absolute value of the effects. The geometries of electromagnet can be defined by seven parameters, as shown in Table 1 and Figure 8. The most effective design variables of normal force and weight are *dv_7_*, *dv_1_* according to the Pareto chart in Figure 10.

**Figure 11.**
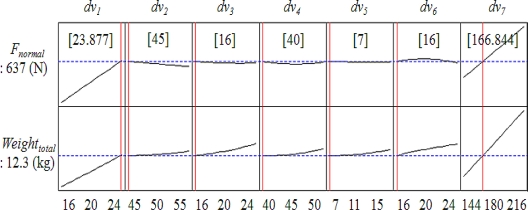
Response optimization.

The values of ineffective design variables are determined by RSM. Figure 11 shows response optimization to find optimal solution according to response curves. The slope of the response function in Figure 11 shows the sensitivity of design variable. According to the response optimization the most sensitive design variables of normal force and weight are *dv_7_*, *dv_1_*.

### 4.3. Kriging Interpolation Method

The purpose of this paper is to minimize the objective function (*Weight_total_*) with the constraint of normal force (*F_normal_*). The kriging interpolation method, the optimum parameters of *θ_1_*, …, *θ_7_* are determined by solving Equation (6). Then, the estimator β is calculated. In Tables 3 and 4, the optimal point is searched to find the point of less than 11.412% of the weight and greater than 7.754% of the normal force of the initially designed electromagnet in the magnetic levitation system. The optimum estimators are shown as Table 4. The optimum solution is the same in the RSM and kriging interpolation method. The reason is that the optimum solution is near the boundary value. However, response values, *Weight_total_* and *F_normal_* by RSM and kriging method, respectively, are different. The simulation result of the predicted optimum set is shown Table 5, displaying good agreement.

## 5. Conclusions

This paper deals with optimum design of a lightweight levitation electromagnet on a vehicle, which also provides passive guide force, in a magnetic levitation system for contactless delivery applications. The optimum design procedure is introduced to design of electromagnet in the magnetic levitation system to reduce its weight and to improve the normal force of the initially designed electromagnet in the magnetic levitation system using several design variables. The most effective design variables are extracted by Pareto chart. The most desired set is determined by RSM and the kriging interpolation method and the influence of each design variables on the objective function can be obtained. This can efficiently increase the precision of the optimization and reduce the number of experiments in the optimization design using the proposed methodologies.

## Figures and Tables

**Table 1.** Design variable and level.

Design variable Level	*dv_1_*	*dv_2_*	*dv_3_*	*dv_4_*	*dv_5_*	*dv_6_*	*dv_7_*
−1	16	45	16	40	7	16	144
0	20	50	20	45	11	20	180
1	24	55	24	50	15	24	216

**Table 2.** Table of mixed orthogonal array *L_18_(2^1^ × 3^7^).*

**Exp.**	***dv_1_***	***dv_2_***	***dv_3_***	***dv_4_***	***dv_5_***	***dv_6_***	***dv_7_***	**Normal force (N)**	**Weight (kg)**

1	16	45	16	40	7	16	144	382.10	8.706
2	16	50	20	45	11	20	180	473.67	12.153
3	16	55	24	50	15	24	216	564.15	16.366
4	20	45	16	45	11	24	216	682.56	15.433
5	20	50	20	50	15	16	144	450.22	10.979
6	20	55	24	40	7	20	180	570.56	13.699
7	24	45	20	40	15	20	216	795.40	16.803
8	24	50	24	45	7	24	144	531.60	12.66
9	24	55	16	50	11	16	180	651.24	14.064
10	16	45	24	50	11	20	144	379.41	10.277
11	16	50	16	40	15	24	180	473.76	12.094
12	16	55	20	45	7	16	216	568.30	13.874
13	20	45	20	50	7	24	180	570.38	13.637
14	20	50	24	40	11	16	216	682.15	15.453
15	20	55	16	45	15	20	144	450.16	10.946
16	24	45	24	45	15	16	180	659.92	14.604
17	24	50	16	50	7	20	216	789.93	16.646
18	24	55	20	40	11	24	144	528.87	12.400

**Table 3.** Optimum level and size.

	
	**Design variable**						

**Model**	***dv_1_***	***dv_2_***	***dv_3_***	***dv_4_***	***dv_5_***	***dv_6_***	***dv_7_***
Initial	20	50	20	40	15	20	180
Optimum (RSM)	23.877	45	16	40	7	16	166.844
Optimum (Kriging)	23.877	45	16	40	7	16	166.844

**Table 4.** Optimum β and correlation parameters for kriging models (N_s_=100).

**Response**	**Correlation parameter (corresponding design variable)**							
	***θ_1_* (*dv_1_)***	***θ_2_* (*dv_2_)***	***θ_3_* (*dv_3_)***	***θ_4_* (*dv_4_)***	***θ_5_* (*dv_5_)***	***θ_6_* (*dv_6_)***	***θ_7_* (*dv_7_)***	***β***
*Weight_total_*	3.026e-3	2.304e-4	0.909e-3	0.201e-3	6.318e-5	1.195e-3	1.289e-2	16.0023
*F_normal_*	1.319e-2	1.382e-5	3.437e-6	1.172e-5	7.819e-6	2.320e-6	4.087e-2	587.009

**Table 5.** Comparison of initial and optimum model.

**Model**		**Weight (kg)**	**Normal force (N)**
Initial	2D FEM	13.319	578.5
	3D FEM	13.319	573.48
	Error(2D *vs.* 3D) %	0	0.86
Optimum	**RSM** (predicted)	12.300	637
	**Kriging method** (predicted)	12.107	611.68
	FEM (verification)	11.799	611.70
	Error (**RSM***vs.* FEM) %	−4.073	−3.972
	Error (**Kriging***vs.* FEM) %	−2.610	0.003
Variation between initial and optimum FEM %		−11.412	7.754
